# Garre's Osteomyelitis of the Mandible Caused by Infected Tooth

**DOI:** 10.1155/2018/1409539

**Published:** 2018-07-08

**Authors:** Hayati Murat Akgül, Fatma Çağlayan, Sevcihan Günen Yılmaz, Gözde Derindağ

**Affiliations:** ^1^Department of Oral and Maxillofacial Radiology, Faculty of Dentistry, Ataturk University, Erzurum, Turkey; ^2^Department of Oral and Maxillofacial Radiology, Faculty of Dentistry, Akdeniz University, Antalya, Turkey

## Abstract

**Aim:**

Garre's osteomyelitis is a local thickening of the periosteum caused by a slight irritation or infection. We aimed to present the extraoral, intraoral, and radiographic findings and postoperative pursuits of two patients diagnosed with Garre's osteomyelitis. In this case report, although clinical findings indicate infection source, these clinical findings are strongly supported by cone-beam computed tomography images. In addition, it can be seen that when we have followed the case I, we have chosen the right path in treatment.

**Case Reports:**

Two patients presented to our clinic due to severe swelling and facial asymmetry in the right and left mandibular region. As a result of the clinical and radiological examinations, the patients were diagnosed with Garre's osteomyelitis. Infected teeth that were responsible for the formation of Garre's osteomyelitis were extracted under antibiotic treatment in both cases. A complete improvement in postoperative control was observed in case I. On the other hand, the other case could not be followed up postoperatively.

**Conclusion:**

In Garre's osteomyelitis, new bone formation can occur in many pathological conditions. Therefore, it should be distinguished from other pathologies that cause new bone formation, such as Ewing's sarcoma, Caffey disease, and fibrous dysplasia.

## 1. Introduction

Garre's osteomyelitis, which was first described by Carl Garre in 1893, is a chronic nonsuppurative sclerotic bone inflammation characterized by a rigid bony swelling at the periphery of the jaw [1–4]. It is most commonly seen in men aged below 30 years [1, 2, 5, 6]. The mandible is more often affected than the maxilla, and it is most generally seen at the lower margin of the mandible in the mandibular first molar region [1, 3, 4, 6, 7]. There is typically a nontender swelling on the medial and lateral sides of the jaw [1, 5, 8, 9]. The size of the swelling may vary from 1-2 cm to the involvement of the entire length of the jaw on the affected side; the thickness of the cortex can reach 2-3 cm [1].

Clinically, Garre's osteomyelitis results in facial asymmetry, since the lesion unilaterally expands to the outer surface of the bone [3–5, 8, 9]. Pain is not a characteristic finding, although severe pain can occur if the lesion is secondarily infected [1, 6]. While it is referred to as nonsuppurative, Garre's osteomyelitis has sometimes been seen to result in a fistula on the skin [3, 6]. The other symptoms are fever, lymphadenopathy, and leukocytosis [1, 3].

There is no macroscopically suppurative lithic area in cases of Garre's osteomyelitis, although histopathological examinations have detected microabscesses and microsequesters [7, 10].

The radiographic appearance varies with the duration of the lesion and the degree of calcification. During the early period, a thin crust-like convex layer appears over the cortex. As the event continues, the cortex is thickened as a result of successive new bone deposits. This lamellar structure is referred to as “onion skin” on radiographs [1, 2, 6, 7]. The adjacent spongiosa bone may exhibit a mixed structure, with some osteolytic areas within the sclerotic field, normal, or sclerotic area [1].

We aimed to present the extraoral, intraoral, and radiographic findings and postoperative pursuits of two patients diagnosed with Garre's osteomyelitis.

## 2. Case Reports

### 2.1. Case I

**Figure 1:**
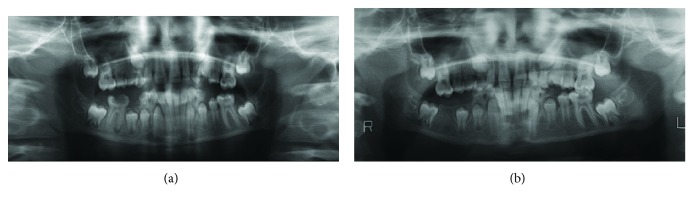
Orthopantomographic image showing a deep caries cavity in the right mandibular first molar tooth, a radiolucent area in its mesial root, and subperiosteal new bone formation below the lower border of the mandible (a). Orthopantomographic image taken four months after tooth extraction showing the return of normal bone contours (b).

**Figure 2:**
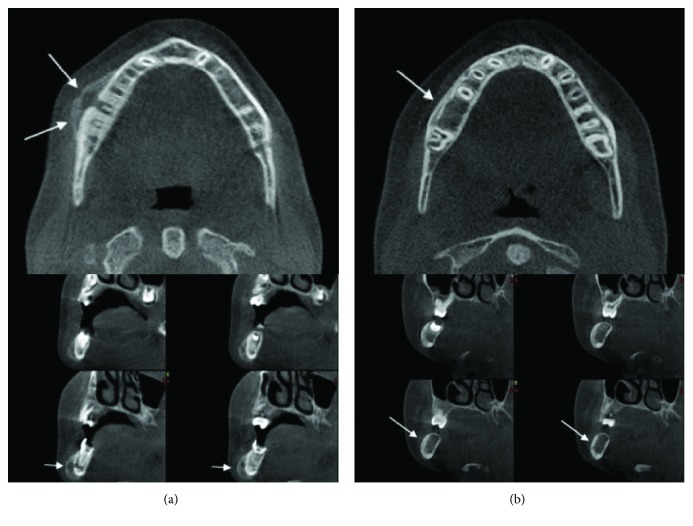
Axial and cross sections in CBCT showing new bone formation and a tunnel-like defect in the vestibule cortical surface of the inflamed bone starting from the apical region of tooth number 46 (a). CBCT image showing decreased cortical bone thickness and the presence of the original cortex within the enlarged portion of the jaw in the postoperative control (b).

Our patient, an eight-year-old girl, presented to our clinic, with severe swelling and facial asymmetry on the right mandibular molar region. We were informed that the patient developed the swelling as a result of an infection three months previously. The patient had been treated with antibiotics, but as that treatment had not proved successful, she was referred to our clinic. In addition, a passed or congenital disease was not specified in the patient's medical history. Clinical examination revealed severe swelling without fluctuation upon palpation and submandibular lymphadenopathy in the right mandibular region. The patient's skin was of normal color and appearance. In the oral examination, the right mandibular first molar tooth was found to have a deep caries cavity and to not be mobile. The other parts of the oral mucosa were normal. The radiographic examination revealed a deep caries cavity and a radiolucent area in the apical region of the right mandibular first molar tooth. There was also a lamellar appearance on the external cortical surface of the mandible as well as at the lower edge of the mandibular corpus, showing focal new bone formation (Figure 1(a)). When the axial and cross sections were evaluated during the examination with cone-beam computed tomography (CBCT), a tunnel-like defect was identified in the cortical bone in the vestibule surface of the inflamed bone, starting from the apical region of the right mandibular first molar tooth. Bone deposition at the radiolucent area in the center was observed at the lower edge of the mandible as well as the vestibule surface in this region (Figure 2(a)). When all these findings were evaluated, it was concluded that the pathologic lesion was Garre's osteomyelitis due to the periapical infection of the right mandibular first molar tooth. In this case, endodontic treatment was considered primarily to retain the infected tooth in the mouth. However, as the patient had come from a remote rural area and could not accept such a treatment due to the prohibitive cost, she was transferred to the surgical clinic, where the most appropriate treatment method was considered to be dental extraction.

The postoperative examination four months later revealed that the bone contours had returned to normal, the asymmetry of the face had disappeared, and the cortical bone thickness had decreased and been remodeled to the previous normal appearance (Figures 1(b) and 2(b)).

### 2.2. Case II

**Figure 3:**
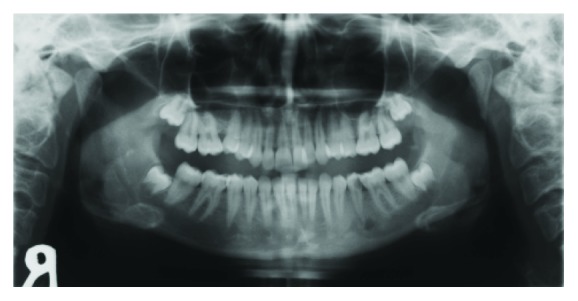
Orthopantomographic image showing a deep caries cavity in the left mandibular second premolar tooth and a radiolucent area in its apical region.

**Figure 4:**
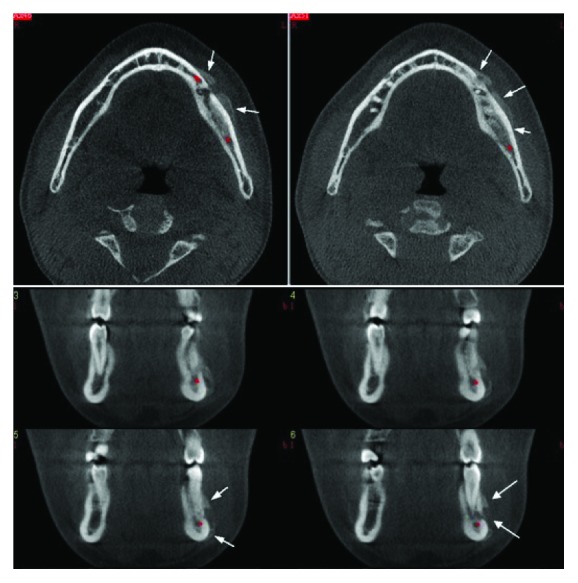
Axial and cross sections showing horizontal bone deposition on the vestibule surface of the mandible.

A 16-year-old girl similarly presented to our clinic with severe swelling and facial asymmetry in the left mandibular premolar region. No pathology could be determined from her clinical and medical history. Clinical examination revealed severe swelling without fluctuation upon palpation, submandibular lymphadenopathy, and a deep caries cavity in the left mandibular second premolar tooth. Additionally, in the radiologic examination, a deep caries cavity was found in the left mandibular second premolar tooth, while a radiolucent area was found in its apical region. However, no change could be detected at the lower edge of the mandibular corpus on these conventional radiographs (Figure 3). For this reason, a sectional examination using CBCT was required. When the axial and coronal sections were evaluated, in addition to the inflammation in the apical region of this tooth, bone deposition was observed horizontally on the vestibule surface of the mandible (Figure 4). When all these findings were evaluated, it was concluded that the pathologic lesion was Garre's osteomyelitis due to the periapical infection of the left mandibular second premolar tooth. Considering the age of the patient, endodontic treatment was considered to retain the infected tooth in the mouth. However, since the patient refused that treatment for similar reasons as in the previous case, the patient was sent to the surgical clinic. Although we wanted her to return to our clinic for a postoperative check-up a few months after the tooth extraction, we were unable to contact her again.

## 3. Discussion

Garre's osteomyelitis is a localized periosteal thickening caused by mild irritation or infection [1, 4, 9, 11]. Although it is sometimes idiopathic, it is known that a moderate infection (such as dental decay, periodontal disease, or soft tissue disease), starting from the spongiosa layer of the jaw and extending into the periosteum, is the result of stimulating bone formation. However, in order for this pathological condition to occur, the balance between the virulent bacteria and oral flora must be impaired, while the periosteal osteoblastic activity must also be high [1, 12].

There is no need for a biopsy during the diagnosis of Garre's osteomyelitis, except the cause is unknown [4, 6]. Conventional radiographic methods or CT images are sufficient for diagnosis [3, 4, 9, 10]. As our two cases exhibited obvious clinical and radiographic features, a biopsy was not required.

In addition to Garre's osteomyelitis, new bone formation can occur in many pathological conditions. Therefore, it should be distinguished from other pathologies that cause new bone formation, including Ewing's sarcoma, Caffey disease, fibrous dysplasia, Paget's disease, osteosarcoma, and hard, nodular, or pedunculated masses seen in the mandible (peripheral osteomas, torus and exostoses, ossifying subperiosteal hematoma, etc.) [3, 4, 6, 10].

Caffey disease presents in a similar view to Garre's osteomyelitis due to the “onion skin” appearance in the bone. However, Caffey disease is distinguished from Garre's osteomyelitis due to the early age of onset (prior to two years of age), it is being more common in the ramus and angulus region of the mandible with bilateral involvement and occurrence in multiple bones [1].

Ewing's sarcoma is similar to Garre's osteomyelitis in terms of the subperiosteal bone formation and appearance in young people. However, Ewing's sarcoma can also be distinguished from Garre's osteomyelitis due to producing osteophytes with a “sun ray” appearance, causing bone enlargement too rapidly and causing more osteolytic reactions in the bone, as well as the occurrence of frequent complications such as facial neuralgia and lip paresthesia [1, 10].

Osteosarcoma can also produce a hard bone mass on the bone surface. However, it is distinguished from Garre's osteomyelitis due to showing the characteristic features of malign tumors, such as new bone formation with a “sun ray” appearance and periosteal reactions in the form of a Codman triangle in radiography [1, 12].

Another pathologic condition requiring a differential diagnosis is fibrous dysplasia. Fibrous dysplasia is seen at younger ages, which is similar to Garre's osteomyelitis, and the resulting bone mass is similar in both shape and volume. Yet, fibrous dysplasia is distinguished from Garre's osteomyelitis due to the “ground glass appearance” as well as the thinning seen in the cortex. Further, unlike Garre's osteomyelitis, it is not associated with any dental infection. In addition, the enlargement is seen in the internal structure of the bone in fibrous dysplasia, whereas the enlargement of the bone in Garre's osteomyelitis is seen on the outer surface of the cortex, while the presence of the original cortex can be detected within the enlarged portion of the jaw in a careful examination [1, 4, 6, 10].

Hard, nodular, or pedunculated masses, such as peripheral osteomas, torus, and exostosis, are radiographically seen as a dense, uniform radiopaque mass extending outward from the cortex. However, Garre's osteomyelitis has regular contours. The clinical appearance of ossifying subperiosteal hematoma may also be similar to that of Garre's osteomyelitis. However, it does not exhibit uniform radiopacity, but can instead be distinguished by the mottled appearance or trabecular structure and trauma story [1].

Different opinions exist regarding the most appropriate treatment for Garre's osteomyelitis. Although hyperbaric oxygen therapy and endodontic treatment have proved successful, the most commonly accepted treatment is the administration of antibiotics and the extraction of the infected tooth [8, 9]. Considering the difficulties associated with applying endodontic treatments in both our cases, antibiotic therapy and tooth extraction were performed. In the first case, the improvement in the bone contours was confirmed in the control films taken four months after the tooth extraction.
